# Multisystem inflammatory syndrome in children (MIS-C): a mini-review

**DOI:** 10.1186/s12245-021-00373-6

**Published:** 2021-09-10

**Authors:** Martina Giacalone, Eric Scheier, Itai Shavit

**Affiliations:** 1 Department of Emergency Medicine and Trauma Center, Meyer University Children’s Hospital, Florence, Italy; 2 Pediatric Emergency Department, Kaplan Medical Center, Israel; Faculty of Medicine, Hebrew University of Jerusalem, Jerusalem, Israel; 3 Pediatric Emergency Department, Rambam Health Care Campus, Haifa, Israel; Rappaport Faculty of Medicine, Technion-Institute of Technology, Haifa, Israel

**Keywords:** SARS-CoV-2, COVID-19, Inflammatory, Children

## Abstract

Multisystem inflammatory syndrome in children (MIS-C) is a novel, life-threatening hyperinflammatory condition that develops in children a few weeks after infection with severe acute respiratory syndrome coronavirus-2 (SARS-CoV-2). This disease has created a diagnostic challenge due to overlap with Kawasaki disease (KD) and KD shock syndrome. The majority of patients with MIS-C present with the involvement of at least four organ systems, and all have evidence of a marked inflammatory state. Most patients show an increase in the level of at least four inflammatory markers (C-reactive protein, neutrophil count, ferritin, procalcitonin, fibrinogen, interleukin-6, and triglycerides). Therapy is primarily with immunomodulators, suggesting that the disease is driven by post-infectious immune dysregulation. Most patients, even those with severe cardiovascular involvement, recover without sequelae. Since coronary aneurysms have been reported, echocardiographic follow-up is needed.

Further study is needed to create uniform diagnostic criteria, therapy, and follow-up protocols.

## Background

A large percentage of children infected with the severe acute respiratory syndrome coronavirus-2 (SARS-CoV-2) are asymptomatic or mildly symptomatic [1].

In April 2020, a novel life-threatening hyperinflammatory condition named multisystem inflammatory syndrome in children (MIS-C) as a complication of SARS-CoV-2 in children was first recognized [2–4].

MIS-C usually develops 4–6 weeks after SARS-CoV-2 infection [4–6], suggesting that the virus may be a trigger for genetically predisposed individuals [2, 7]. Not all patients test positive for SARS-CoV-2 real time-polymerase chain reaction (RT-PCR) on nasal swab, but the majority show serological positivity or an epidemiologic link to SARS-CoV-2 infection [8, 9].

## Case definition (May 2020)

The European and US Centers for Disease Prevention and Control (CDC) criteria for the diagnosis of MIS-C are as follows: age < 21 years, fever ≥ 1 day, laboratory evidence of inflammation, and multisystem (≥ 2) organ involvement (cardiac, renal, respiratory, hematologic, gastrointestinal, dermatologic, or neurological) requiring hospitalization; no alternative plausible diagnoses; and positive for SARS-CoV-2 infection by RT-PCR, serology, or antigen test or exposure to suspected or confirmed SARS-CoV-2 infection within the 4 weeks prior to the onset of symptoms [4]. The World Health Organization (WHO) criteria are somehow different: 0–19 years of age with fever ≥ 3 days, multisystem (≥ 2) organ involvement (mucocutaneous, hypotension/shock, cardiac, gastrointestinal), and no microbial cause of inflammation, including bacterial sepsis, staphylococcal, or streptococcal shock syndromes. The SARS-CoV-2 epidemiologic link is based on RT-PCR, antigen test, or serology positive or likely contact with patients with SARS-CoV-2 [10].

## Clinical presentation

The median age of MIS-C patients is usually 8–9 years, and the majority of the patients have no preexisting medical conditions [2–13]. In most reported cases, the patients were males (57%), and many cases were reported in Hispanic and non-Hispanic Black children [12, 13]. Most patients (71–90%) presented with the involvement of at least four organ systems, and over half of the patients required admission to an intensive care unit during their hospital stay [5, 8, 12].

Various signs and symptoms have been described, most commonly fever > 38 °C (100%), abdominal pain, vomiting or diarrhea (53–92%), erythematous skin rash (52–62%), hypotension (49–51%), mucocutaneous involvement (70–74%), and conjunctival changes (45–54%) [3, 5–9, 12]. Other symptoms, such as sore throat, neurologic symptoms (headache, lethargy, or confusion), lymphadenopathy, and edematous hands and feet are variously reported [7]. Respiratory symptoms are also variously reported (30–70%) [5, 12].

Importantly, more than 80% of MIS-C patients present with cardiac involvement. Cardiac dysfunction (28–62%), shock (35–50%), myocarditis (17–22%), and coronary artery dilatation or aneurysm (8–24%), and a small number present with cardiac electrical abnormalities and arrhythmias. Acute kidney injury, usually mild and with rapid recovery, is also described [3, 5–9, 11–13].

## Diagnostic tests and predictors of disease severity

Most patients showed an increase in the level of at least 4 inflammatory markers (C-reactive protein, neutrophil count, ferritin, procalcitonin, fibrinogen, interleukin-6, and triglycerides) [3, 5, 7]. Thrombocytopenia (40%) and lymphopenia (30%) are also reported [12]. Frequently, children with MIS-C show elevated D-dimer levels (90%) [8], but, despite an observed prothrombotic state, thrombosis is rare [12].

Cardiac biomarkers (troponin, brain natriuretic peptide [BNP], or pro-brain BNP [proBNP]) are elevated in a large proportion of patients (64–95% and 73–95%) and indicate cardiac involvement [3, 7, 9, 11]. Cardiac and inflammatory biomarkers not only suggest a diagnosis of MIS-C but may also reflect the severity of illness [9, 11]. A recent study suggests that high ED levels of proBNP can serve as early warning indicators for the requirement of inotropic/vasoactive support in MIS-C [14]. Point-of-care ultrasound or complete echocardiography may be useful to promptly recognize patients with cardiac dysfunction and to adjust therapy or to evaluate for alternate diagnoses [3, 9]. Other imaging tests should be guided by clinical judgment, and repeating the laboratory evaluation during the course of illness may be useful to identify patients at risk for deterioration.

## Comparison of MIS-C and similar syndromes

The clinical overlap of SARS-CoV-2, MIS-C, and Kawasaki disease (KD) creates a formidable diagnostic challenge.

### MIS-C and SARS-CoV-2

Patients with MIS-C, compared to SARS-CoV-2 patients, are younger and less likely to have one or more underling medical conditions. Presenting symptoms and signs are similar among the two groups with the exception of mucocutaneous findings. Patients with MIS-C are more likely to have both cardiovascular and mucocutaneous involvement, and the majority test positive for SARS-CoV-2 antibody. MIS-C patients have a higher median neutrophil-to-lymphocyte ratio and higher CRP and more frequently have thrombocytopenia [5–9, 11–13].

### MIS-C, atypical KD, and KD shock syndrome (KDSS)

KD generally presents in children less than 8 years old and is more common in Asians [6]. MIS-C presents with a greater variety of signs and symptoms and is more often characterized by gastrointestinal and neurologic symptoms. Children with MIS-C have lower absolute lymphocyte and platelet counts, higher ferritin and D-dimer levels, and a higher likelihood of having elevated troponin or ProBNP levels [7].

The typical cardiac features in the two syndromes differ: MIS-C more typically presents with ventricular dysfunction and shock (more than 50% in MIS-C versus 5–10% in KD) [5]. The development of coronary artery aneurysms in both disorders may increase diagnostic uncertainty [7].

## Treatment

Due to lack of evidence, published guidelines are based on expert consensus.

Supportive care with fluid resuscitation, inotropes (required by 40–48%), mechanical ventilation (required by 20–47%, often as a result of cardiovascular collapse), and, in the most severe cases, extracorporeal membrane oxygenation (ECMO) support may be required in the acute phase [3, 5, 7–9].

Therapy for MIS-C is primarily with immunomodulators, suggesting that the disease is driven by post-infectious immune dysregulation [2]. Many children were successfully treated with high dose intravenous gamma globulins (IVIG) alone or combined with corticosteroid. These regimens were adopted from KD guidelines, and combining IVIG with corticosteroid led to a lower rate of treatment failure [5, 7]. Treatment with IVIG and methylprednisolone vs IVIG alone was associated with a more favorable fever course [15]. Additional first-line therapies included antiplatelet medications (58–74%) and anticoagulants (37–44%) [5, 7–9]. Other medications were also reported in case series of patients with MIS-C: interleukin-6 inhibitors (8%), anti-tumor necrosis factor (5%), and interleukin-1Ra inhibitor (2–13%) [5, 7–9].

## Prognosis and follow-up

The majority of MIS-C patients, even after critical care and with severe cardiovascular involvement, recovered without sequelae (70–97%) [5, 9, 11, 15]. Since coronary aneurysms have been reported to develop in the convalescent phase of illness, echocardiographic follow-up is needed, even in patients with no cardiac involvement in the acute phase of illness [7]. Until more is known about long-term morbidity, it is reasonable to use KD guidelines to guide outpatient follow-up [5, 7].

## Conclusion

MIS-C, a SARS-CoV-2-related condition, is a novel pediatric syndrome characterized by the presence of fever, elevated inflammatory markers, and multi-organ involvement. Clinical characteristics are shared to some extent with KD, and therapy is guided by KD guidelines. Despite a high level of morbidity, the majority of patients experience a good outcome. As this is a new pediatric syndrome, much is yet to be learned about long-term prognosis, and further research is needed to create uniform diagnostic criteria and to optimize therapy protocols.

## Data Availability

All data analyzed in this study are included in this published article**.**

## Abbreviations

COVID 19: Coronavirus disease 2019
SARS-CoV-2: Severe acute respiratory syndrome
MIS-C: Multisystem inflammatory syndrome in children
RT-PCR: Real-time polymerase chain reaction
CDC: Centers for Disease Prevention and Control
WHO: World Health Organization
BNP: Brain natriuretic peptide
KD: Kawasaki disease
KDSS: Kawasaki disease shock syndrome
ECMO: Extracorporeal membrane oxygenation
IVIG: Intravenous gamma globulins

## References

[1] DeBiasi RL, Delaney M (2021). Symptomatic and asymptomatic viral shedding in pediatric patients infected with severe acute respiratory syndrome coronavirus 2 (SARS-CoV-2): Under the Surface. JAMA Pediatr..

[2] Vogel TP, Top KA, Karatzios C, Hilmers DC, Tapia LI, Moceri P, Giovannini-Chami L, Wood N, Chandler RE, Klein NP, Schlaudecker EP, Poli MC, Muscal E, Munoz FM (2021). Multisystem inflammatory syndrome in children and adults (MIS-C/A): case definition & guidelines for data collection, analysis, and presentation of immunization safety data. Vaccine.

[3] Riphagen S, Gomez X, Gonzalez-Martinez C, Wilkinson N, Theocharis P (2020). (2020) Hyperinflammatory shock in children during COVID-19 pandemic. Lancet.

[4] Center for Disease Control and Prevention. Information for healthcare providers about multisystem inflammatory syndrome in children (MIS-C). Available via https://www.cdc.gov/mis-c/hcp/. Accessed 5 Aug 2021

[5] Feldstein LR, Rose EB, Horwitz SM, Collins JP, Newhams MM, Son MBF, Newburger JW, Kleinman LC, Heidemann SM, Martin AA, Singh AR, Li S, Tarquinio KM, Jaggi P, Oster ME, Zackai SP, Gillen J, Ratner AJ, Walsh RF, Fitzgerald JC, Keenaghan MA, Alharash H, Doymaz S, Clouser KN, Giuliano JS Jr, Gupta A, Parker RM, Maddux AB, Havalad V, Ramsingh S, Bukulmez H, Bradford TT, Smith LS, Tenforde MW, Carroll CL, Riggs BJ, Gertz SJ, Daube A, Lansell A, Coronado Munoz A, Hobbs CV, Marohn KL, Halasa NB, Patel MM, Randolph AG, Overcoming COVID-19 Investigators, CDC COVID-19 Response Team (2020). Multisystem inflammatory syndrome in U.S. children and adolescents. N Engl J Med.

[6] Belhadjer Z, Méot M, Bajolle F, Khraiche D, Legendre A, Abakka S, Auriau J, Grimaud M, Oualha M, Beghetti M, Wacker J, Ovaert C, Hascoet S, Selegny M, Malekzadeh-Milani S, Maltret A, Bosser G, Giroux N, Bonnemains L, Bordet J, di Filippo S, Mauran P, Falcon-Eicher S, Thambo JB, Lefort B, Moceri P, Houyel L, Renolleau S, Bonnet D (2020). Acute heart failure in multisystem inflammatory syndrome in children in the context of global SARS-CoV-2 pandemic. Circulation.

[7] Whittaker E, Bamford A, Kenny J, Kaforou M, Jones CE, Shah P, Ramnarayan P, Fraisse A, Miller O, Davies P, Kucera F, Brierley J, McDougall M, Carter M, Tremoulet A, Shimizu C, Herberg J, Burns JC, Lyall H, Levin M, for the PIMS-TS Study Group and EUCLIDS and PERFORM Consortia (2020). Clinical characteristics of 58 children with a pediatric inflammatory multisystem syndrome temporally associated with SARS-CoV-2. JAMA.

[8] Godfred-Cato S, Bryant B, Leung JW, Oster ME, Conklin L, Abrams J, Roguski K, Wallace B, Prezzato E, Koumans EH, Lee EH, Geevarughese A, Lash MK, Reilly KH, Pulver WP, Thomas D, Feder KA, Hsu KK, Plipat N, Richardson G, Reid H, Lim S, Schmitz A, Pierce T, Hrapcak S, Datta D, Morris SB, Clarke K, Belay E, California MIS-C Response Team (2020). MIS-C Response Team On. COVID-19–associated multisystem inflammatory syndrome in children —United States, March–July 2020. MMWR Morb Mortal Wkly Rep.

[9] Valverde I, Singh Y, Sanchez-de-Toledo J, Theocharis P, Chikermane A, di Filippo S, Kuciñska B, Mannarino S, Tamariz-Martel A, Gutierrez-Larraya F, Soda G, Vandekerckhove K, Gonzalez-Barlatay F, McMahon CJ, Marcora S, Napoleone CP, Duong P, Tuo G, Deri A, Nepali G, Ilina M, Ciliberti P, Miller O, Iriart X, Hubrechts J, Kuipers IM, Sousa AR, Donti A, Sharpe A, Reinhardt Z, Cairello F, de Wolf D, Vieira M, Lazea C, Gran F, Medrano-Lopez C, Ortiz-Garrido A, Vukomanovic V, Brent BE, Milanesi O, Dewals W, Manso B, Valsangiacomo-Buchel E, Francisco A, Seghaye MC, Loeckx I, Rodriguez-Gonzalez M, ReyGarcía SM, Ziesenitz VC, Bordin G, Doros G, Grangl G, Fadl SU, Perminow KV, Centeno F, Pinto F, Niemelä J, Kanthimathinathan HK, Randanne PC, Niszczota C, Zuccotti GV, Gordillo IL, Obeyasekhara M, Armstrong C, Butler K, Ciuffreda M, Villar AM, Pappula N, Caorsi R, Singh D, Durairaj S, McLeod K, Calcagni G, Quizad Y, Gewillig M, Kuijpers TW, Ataide R, Fabi M, Bharucha T, Abbas K, Magrass SA, Wong J, Iacob D, Balcells J, GilVillanueva N, Cuenca-Peiro V, Cerovi I, Sarfatt A, Zaqout M, Sanchez-Valderrabanos E, Kelly-Geyer J, Diogo F, Cajgfinger N, Françoise M, Rueda-Nuñez F, Gorenflo M, Grison A, Mihailov D, Koestenberger M, Alcalde C, Trigo C, Arola A, Hanseus K (2021). Acute cardiovascular manifestations in 286 children with multisystem inflammatory syndrome associated with COVID-19 infection in Europe. Circulation.

[10] World Health Organization. Multisystem inflammatory syndrome in children and adolescents temporally related to COVID-19: Scientific Brief. Available via https://www.who.int/news-room/commentaries/detail/multisystem-inflammatory-syndrome-in-children-and-adolescents-with-covid-19. Accessed 5 Aug 2021

[11] Abrams JY, Oster ME, Godfred-Cato SE, Bryant B, Datta SD, Campbell AP, Leung JW, Tsang CA, Pierce TJ, Kennedy JL, Hammett TA, Belay ED (2021). Factors linked to severe outcomes in multisystem inflammatory syndrome in children (MIS-C) in the USA: a retrospective surveillance study. Lancet Child Adolesc Health.

[12] Belay ED, Abrams J, Oster ME, Giovanni J, Pierce T, Meng L, Prezzato E, Balachandran N, Openshaw JJ, Rosen HE, Kim M, Richardson G, Hand J, Tobin-D’Angelo M, Wilson S, Hartley A, Jones C, Kolsin J, Mohamed H, Colles Z, Hammett T, Patel P, Stierman B, Campbell AP, Godfred-Cato S (2021). Trends in geographic and temporal distribution of US children with multisystem inflammatory syndrome during the COVID-19 pandemic. JAMA Pediatr..

[13] Feldstein LR, Tenforde MW, Friedman KG, Newhams M, Rose EB, Dapul H, Soma VL, Maddux AB, Mourani PM, Bowens C, Maamari M, Hall MW, Riggs BJ, Giuliano JS, Singh AR, Li S, Kong M, Schuster JE, McLaughlin GE, Schwartz SP, Walker TC, Loftis LL, Hobbs CV, Halasa NB, Doymaz S, Babbitt CJ, Hume JR, Gertz SJ, Irby K, Clouser KN, Cvijanovich NZ, Bradford TT, Smith LS, Heidemann SM, Zackai SP, Wellnitz K, Nofziger RA, Horwitz SM, Carroll RW, Rowan CM, Tarquinio KM, Mack EH, Fitzgerald JC, Coates BM, Jackson AM, Young CC, Son MBF, Patel MM, Newburger JW, Randolph AG, Overcoming COVID-19 Investigators (2021). Characteristics and outcomes of US children and adolescents with multisystem inflammatory syndrome in children (MIS-C) compared with severe acute COVID-19. JAMA.

[14] Tibi R, Hadash A, Khoury A, Butbul-Aviel Y, Ben-Ari J, Shavit I. Emergency department levels of NT-proBNP and inotropic/vasoactive support in multi-inflammatory syndrome in children (MIS-C). Am J Emerg Med. 2021. 10.1016/j.ajem.2021.07.046. 10.1016/j.ajem.2021.07.046 PMC8316715 34334284

[15] Ouldali N, Toubiana J, Antona D, Javouhey E, Madhi F, Lorrot M, Léger PL, Galeotti C, Claude C, Wiedemann A, Lachaume N, Ovaert C, Dumortier M, Kahn JE, Mandelcwajg A, Percheron L, Biot B, Bordet J, Girardin ML, Yang DD, Grimaud M, Oualha M, Allali S, Bajolle F, Beyler C, Meinzer U, Levy M, Paulet AM, Levy C, Cohen R, Belot A, Angoulvant F, Rames C, Donzeau A, Lety S, Fedorczuk C, Lajus M, Bensaid P, Laoudi Y, Pons C, Beaucourt C, de Pontual L, Aupiais C, Lefevre-Utile A, Richard M, Goisque E, Iriart X, Brissaud O, Bailhache M, Segretin P, Molimard J, Orcel MC, Benoist G, Amouyal E, Guerder M, Pouyau R, de Guillebon de Resnes JM, Mezgueldi E, Cour-Andlauer F, Horvat C, Poinsot P, Frachette C, Ouziel A, Gillet Y, Barrey C, Brouard J, Faucon C, Ginies H, Ro V, Elanga N, Gajdos V, Basmaci R, Danekova N, Mutar H, Rouget S, Torterüe X, Nattes E, Hau I, Biscardi S, el Jurdi H, Jung C, Epaud R, Delestrain C, Carlier-Gonod A, Chavy C, Colomb B, Litzler-Renault S, Semama D, Huet F, Sarakbi M, Mortamet G, Bost-Bru C, Kevorkian-Verguet C, Lachaud M, Vinit C, Hentgen V, Leroux P, Bertrand V, Parrod C, Craiu I, Kone-Paut I, Durand P, Tissiere P, Morin L, Miatello J, Morelle G, Guiddir T, Borocco C, Guillot C, Leteurtre S, Dubos F, Jouancastay M, Recher M, Martinot A, Voeusler V, Languepin J, Morand A, Bosdure E, Bresson V, Vanel N, Ughetto F, Michel F, Marie C, Blonde R, Nguyen J, Garrec N, Chalvon-Demersay A, Masserot-Lureau C, Colas AS, Ferrua C, Larakeb A, Benkaddouss S, Mathivon L, Monfort M, Naji S, Carbasse A, Milesi C, Schweitzer C, Giroux N, Boussard N, Romefort B, Launay E, Gras-le Guen C, Ali A, Blot N, Tran A, Rancurel A, Haas H, Afanetti M, Bernardor J, Talmud D, Jhaouat I, Monceaux F, Chosidow A, Romain AS, Grimprel E, Rambaud J, Jean S, Starck J, Levy Y, Guedj R, Carbajal R, Parisot P, Poncelet G, Wolff R, Lacarra B, Maroni A, Naudin J, Geslin G, Maurice L, Deho A, Lebourgeois F, Chomton M, Dauger S, Genuini M, Benzouid C, Lokmer J, Bonnefoy R, Melki I, Dingulu G, Gaschignard J, Ducrocq C, Pouletty M, Corseri O, Faye A, Rybak A, Titomanlio L, Hurtaux MF, Garcelain G, Bonacorsi S, Bidet P, Birgy A, Renolleau S, Lesage F, Moulin F, Dupic L, de Saint Blanquat L, Heilbronner C, Vedrenne-Cloquet M, Salvador E, Bendavid M, de Marcellus C, Chareyre J, Pinhas Y, Brisse J, Taylor M, Debray A, Adnot P, Chalumeau M, Abadie V, Frange P, Cohen JF, Curtis W, Chappuy H, Belhadjer Z, Auriau J, Méot M, Houyel L, Bonnet D, Delacourt C, Drummond D, Bader-Meunier B, Quartier P, Delion F, Blanc P, Caron E, Maledon N, Robert B, Pantalone L, Kouider H, Loeile C, Loron G, Vittot C, Blanc T, Pinquier D, Buisson F, Flodrops H, Karim JB, Sarton R, Mokraoui F, Escoda S, Deschamps N, Bonnemains L, Mahi SL, Mertes C, Terzic J, Idier C, Benezech A, Simon T, Decramer S, Karsenty C, Brehin C, Chenichene S, Ursulescu NM, Manteau C, Delattre M, Dalichoux B, French Covid-19 Paediatric Inflammation Consortium (2021). Association of intravenous immunoglobulins plus methylprednisolone vs immunoglobulins alone with course of fever in multisystem inflammatory syndrome in children. JAMA.

